# Common neural value representations of hedonic and utilitarian products in the ventral striatum: An fMRI study

**DOI:** 10.1038/s41598-019-52159-9

**Published:** 2019-10-30

**Authors:** Kosuke Motoki, Motoaki Sugiura, Ryuta Kawashima

**Affiliations:** 1 Department of Food Management, Miyagi University, Sendai, Japan; 2 Institute of Development, Aging and Cancer, Tohoku University, Sendai, Japan

**Keywords:** Human behaviour, Reward

## Abstract

Hedonic goods are goods that people buy to obtain emotional experiences, such as joy or excitement, while utilitarian goods are bought to meet functional or instrumental needs. Although research in neuroscience suggests that the values of hedonic and utilitarian goods are similarly represented, it remains largely unknown how these values are mapped during purchasing decisions or task-irrelevant judgments. It has been suggested that people rely more on hedonic (vs. utilitarian) factors when making task-irrelevant judgments, and that this is amplified by trait-reward seeking. Functional magnetic resonance imaging (fMRI) can directly measure the mental processes involved in explicit or task-irrelevant value judgments. Using fMRI, we found that the explicit value of hedonic and utilitarian goods was commonly processed in the ventral striatum. In contrast, no significant results were obtained in common neural processing of task-irrelevant hedonic and utilitarian value. Additionally, we did not find any evidence that trait-reward seeking modulates task-irrelevant hedonic (vs. utilitarian) value processing. Our findings show that the value of both hedonic and utilitarian goods is commonly represented in the ventral striatum, and indicate that the value construct underlying consumer purchases is unidimensional.

## Introduction

Both affective and cognitive (functional and instrumental need-related) factors can play a role in purchasing decisions. You may, for example, buy a bar of chocolate because you want to feel happy when eating the delicious chocolate, or you may buy bug killer to avoid negative emotions. In contrast, you may buy a microwave because it is useful rather than for affective gratification; therefore, the decision is cognitively-based. Purchases can also involve both affective and cognitive factors.

The value of goods may be automatically processed. Imagine that you are walking into a shopping mall. You may see many items, including goods you buy for affective reasons (e.g., junk food, movies, or concert tickets), as well as ones that you buy for cognitive reasons (e.g., detergent, a washing machine, or vitamin tablets). You may see and pass almost all of these products without scrutinizing their value. Nevertheless, you may consider the value of goods even though you do not have to do so—perhaps automatically.

The focus of the present research is the role of goods-induced and trait affect in unconscious value processing. Although recent neuroscience findings suggest that the values of hedonic and utilitarian goods are represented in the same brain regions^1^, it remains unknown how these values are mapped during task-irrelevant judgments (when explicit economic evaluations are not required). The affect-cognition theory suggests that affective reactions can be processed faster during automatic judgments, and that affective processing predominates^2^. Moreover, a chronic (i.e., trait) reward orientation is thought to be necessary to generate predominant automatic processing of affective value^3^. However, it remains unknown how consumers automatically value goods based on goods-induced and trait affect.

Recent advances in neuroscience, and particularly in functional magnetic resonance imaging (fMRI), enable researchers to investigate these issues. Neural data captured by fMRI have unique advantages for investigating research questions pertaining to consumer behaviors such as (1) distinguishing among different psychological processes, (2) measuring task-irrelevant consumer behaviors, and (3) understanding the biological origins of individual differences in consumer behaviors^4,5^. fMRI can track mental activity reflected in neural responses in real-time consumer decision processes. The characteristics of fMRI rely less on self-reporting, and reduce concerns about response biases such as social desirability, memory distortion, and fabrication. Additionally, fMRI provides better spatial resolution than many other neuroscientific methods (e.g., EEG, fNIRS, or MEG), and thus it can reliably measure activities in deep brain structures such as value-related mesolimbic regions (e.g., the ventral striatum).

In this study, our goal was to clarify value processing of consumer goods in the brain. We empirically tested how (1) consumers explicitly and task-irrelevantly encode the values of goods with affect-based and cognitively- based appeal and (2) how the related trait (reward sensitivity) modulates the task-irrelevant value signals of affectively- vs. cognitively-based goods using fMRI.

## Theoretical Background

### Hedonic and utilitarian goods

Consumer choices are based on two distinct motives for buying^6^. The value of some products (e.g., chocolate) is hedonically- (i.e., affectively-) based, whereas other products (e.g, microwaves) are valued for utilitarian reasons (i.e., they serve functional and instrumental needs)^2,7^. We refer to the former as hedonic goods and the value they are accorded as hedonic value. Hedonic goods provide emotional experiences such as joy, excitement, fun, and pleasure^6^. Hedonic goods have high levels of hedonic dimension attributes (and lower levels of utilitarian dimension attributes). Hedonic goods include sports cars, fashionable clothes, and action movies. Conversely, we refer to goods purchased because of their utility as utilitarian goods, and the value they are accorded as utilitarian value. Utilitarian goods meet functional and instrumental needs, and their attractive features are more utilitarian than hedonic^6^. Utilitarian goods include products such as microwaves and vacuum cleaners^6^. When people purchase utilitarian goods, they place emphasis on their usefulness, convenience, and functionality, not their affective features.

### Neural common currency hypothesis

A growing body of neuroscience research supports a neural common currency hypothesis. The notion is that specific brain regions in the ventromedial prefrontal cortex (ventromedial prefrontal cortex [PFC] and ventral striatum [VS]) encode consumers’ valuations of different types of goods^1^. vmPFC and VS activity increases when participants view purchased goods, as compared to when they do not^8,9^. In addition, vmPFC activity while viewing goods was positively correlated with the amount of money the consumer was willing to pay for the goods being viewed^10,11^. The neural pattern of the vmPFC also encoded the value of different types of goods^12^. Moreover, meta-analyses have shown that the vmPFC and VS are involved in value-related decisions^13^. Therefore, the vmPFC and VS may track overall stimulus value during a shopping decision. The neural common currency hypothesis suggests that the vmPFC and VS encode all types of value during the purchasing of goods.

H1: Value signals for both hedonic and utilitarian goods are commonly processed in specific brain regions (the vmPFC and VS) during purchasing.

### Task-irrelevant consumer value

For a better understanding of consumer value, we should clarify how hedonic and utilitarian values are affected by explicit vs. task-irrelevant value judgments. Explicit value judgments occur during typical shopping decisions, whereas task-irrelevant value judgments are automatic evaluations. Consumers assess the value of goods even when they do not have to do it^14^. People automatically allocate attention to task-irrelevant items associated with a reward^15^ and, therefore, engage in task-irrelevant economic evaluations—i.e., evaluations not associated with purchasing decisions. However, it remains unknown how the values associated with hedonic and utilitarian goods that are not being considered for purchase are represented.

### Neural common currency hypothesis: Is the task-irrelevant value of hedonic and utilitarian goods assessed along the same dimension?

The neural common currency hypothesis suggests that hedonic and utilitarian values share the same dimension during task-irrelevant value judgments. As with explicit value judgments, the vmPFC may encode both hedonic and utilitarian values during value-irrelevant judgments. A previous study used a passive viewing paradigm, and participants viewed goods in an MRI scanner. The participants then indicated whether they wanted to purchase the goods. vmPFC activity predicted whether participants bought the goods^16^. Another study had participants engage in cognitive tasks in which they were not required to value goods. The results show that valuation regions such as vmPFC and other limbic regions are related to task-irrelevant economic values^17^. Moreover, one study employing various stimuli (e.g., faces, houses, or paintings) shows that activation of the vmPFC, VS, and other regions is positively correlated with the value of stimuli when participants see and rate the age of the stimuli (they did not have to value the goods)^18^. Although these previous studies do not differentiate between hedonic and utilitarian goods, their results suggest that hedonic and utilitarian values are both assessed in value-related regions (vmPFC and VS) during task-irrelevant value judgments.

H2: Value signals of both hedonic and utilitarian goods are commonly processed in specific brain regions (the vmPFC and VS) during value-irrelevant judgments.

### The task-irrelevant value of hedonic (vs. utilitarian) goods may be amplified

Task-irrelevant economic valuations may predominantly occur for hedonic goods^2^. The value of hedonic goods can be based on positive arousal (a combination of positive valence and high arousal), because hedonic attitudes reflect both valence (e.g., fun, enjoyment) and arousal (e.g., excitement, thrilling)^6^. According to the affect-cognition model, affective reactions occur in a relatively automatic manner, even if processing resources are not directed to the task^2^. In contrast, choice of products is not influenced by hedonic features when processing resources are unlimited^2^. The affect-integration-motivation framework also indicates that activity in brain regions related to positive arousal (ventral striatum: VS) occurs first, followed by further value representation (vmPFC). Previous studies show that people allocate their attention to task-irrelevant stimuli with positive arousing features, such as money^15^, emotional pictures^19^, or tasty foods^20^, even when they do not have to focus on the stimuli. These findings indicate that task-irrelevant value signals are more likely to be encoded in the vmPFC and VS for hedonic than for utilitarian goods.

H3: Value signals for hedonic goods are more likely to be encoded in the vmPFC and VS than those for utilitarian goods.

### The task-irrelevant hedonic (vs. utilitarian) value may be amplified by behavioral activation system

The value of task-irrelevant hedonic goods is likely to be amplified by the intensity of approach to rewarding stimuli. The affect-integration-motivation framework indicates that approach motivation mediates the relationship between preceding positive arousal and further evaluations^21^. It has been also shown that positive arousal may be linked to both approach motivation and reward^22^. We propose that individual differences in motivation to approach rewarding cues (i.e., in the Behavioral Activation System [BAS]) moderate the effect of goods type and/or judgment modes on value responsiveness.

Value responsiveness is greater with increased levels of approach motivation when the judgment mode is task-irrelevant (vs. explicit) and goods are hedonic (vs. utilitarian). Gray advocated for a reinforcement sensitivity theory of personality. According to the theory, the BAS encodes individual differences in sensitivity to rewarding stimuli. The BAS regulates approach motivation and goal-directed behavior to attain rewards (e.g., Gray, 1994). The biological substrate of the BAS is thought to comprise brain areas belonging to dopaminergic systems such as the vmPFC and VS. Previous studies suggest that personality traits affect choice of hedonic (vs. utilitarian) goods when the judgment mode is relatively task-irrelevant. Individuals with a more reactive BAS tend to show an automatic processing bias that is susceptible to priming of hedonic goods (i.e., high-fat foods)^3^. Given the role of the BAS in (automatic) affective reactions, the BAS modulates evaluations of hedonic goods more than utilitarian goods during task-irrelevant judgments, but not during explicit judgments.

H4: When value judgments are task-irrelevant (vs. explicit), value responsiveness to hedonic goods (greater activity in the VS and vmPFC) is positively related to BAS reward sensitivity.

### The current study

In this study, we aimed to understand value processing of consumers using fMRI. It remains unknown how consumers explicitly or automatically value goods based on goods-induced and trait affect. Therefore, we investigated how values of hedonic and utilitarian goods are represented in the brain during (1) shopping and (2) task-irrelevant judgments and (3) how trait reward sensitivity (i.e., BAS) modulates this process. Participants performed two decision-making tasks with the same hedonic and utilitarian goods across tasks. They made purchasing decisions (for which values are directly explicit) or perceptual decisions (for which values are unrelated to the purchasing decisions and, therefore, task-irrelevant). This protocol allowed us to investigate the value representations of hedonic and utilitarian goods during task-irrelevant (vs. explicit) judgments, as well as how the BAS modulates task-irrelevant value representations.

## Materials and Methods

### Participants

Participants in this study were 34 healthy, right-handed volunteers with no history of neurological or psychiatric illnesses. As in our previous studies^23,24^, participants’ history of neurological or psychiatric illnesses was obtained through self-reports. Participants were recruited through flyers posted on a university campus and provided their written informed consent after receiving an explanation regarding the nature of the experiment. All procedures were conducted according to the Declaration of Helsinki. The experimental protocol was approved by the ethics committee of Tohoku University School of Medicine.

We excluded seven participants. Four participants dropped out due to excessive head movements (more than 3 mm) during MRI scanning. Three participants were also removed from the analyses because they purchased too few items to provide sufficient data for our model fitting (i.e., they paid 0 yen more than 90 percent of the time for either hedonic or utilitarian goods). Finally, data from 27 participants were used (mean age = 20.37, *SD* = 1.15, 6 females).

### Stimulus

Participants in this neuroimaging study evaluated the cover of books during MRI sessions. We classified books as either hedonic or utilitarian goods (either predominantly hedonic or utilitarian attitude scores). According to consumer literature, hedonic goods have predominantly hedonic attitude scores, and utilitarian goods have predominantly utilitarian attitude scores. Hedonic/utilitarian goods with predominantly hedonic/utilitarian attitude scores are bought for hedonic/utilitarian reasons, respectively^6^.

The hedonic and utilitarian goods used in the MRI experiment were selected from 500 books through a preliminary Internet study. A total of 1,000 participants joined the preliminary Internet study and randomly evaluated each of the 50 books in the pool. Each book was evaluated based on questions, including hedonic and utilitarian scales for consumer attitudes^6^, willingness-to-pay, knowledge content, and brightness of the cover by each of the 100 participants. The hedonic and utilitarian scales of consumer attitudes consist of five items each (hedonic: fun/not fun, exciting/dull, delightful/not delightful, thrilling/not thrilling, enjoyable/unenjoyable; utilitarian: effective/ineffective, helpful/unhelpful, functional/not functional, necessary/unnecessary, practical/impractical). All ratings were conducted on a 7-point Likert scale from 1 (not at all) to 7 (very much). The book prices were in the same range (500–1000 yen corresponding to $4.5–9.0), but participants were told not to consider the price during evaluation.

Finally, 152 books were selected (76 hedonic and 76 utilitarian) as goods in this study based on the hedonic and utilitarian scales of consumer attitudes^6^. We define hedonic goods as having higher hedonic and lower utilitarian attitude scores than utilitarian goods, and *vice versa* for utilitarian goods based on the hedonic and utilitarian scales of consumer attitudes (hedonic attitude: 4.159 ± 0.130 for hedonic goods and 3.819 ± 0.133 for utilitarian goods; utilitarian attitude: 3.402 ± 0.1436 for hedonic goods and 4.518 ± 0.242 for utilitarian goods). The hedonic and utilitarian goods have the same distributions of perceptual levels, to match the total number of people and the lines of the title in the book ranging from 2 to 5 (3.778 ± 1.064 for both hedonic and utilitarian goods).

### Task design

**Figure 1:**
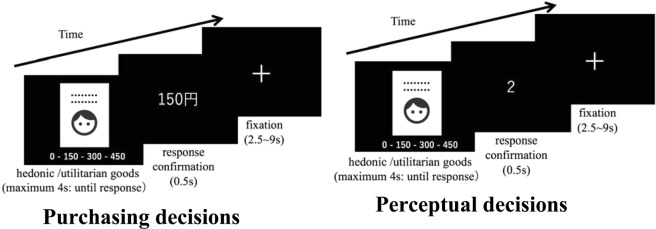
Participants performed two types of task in relation to identical stimuli (book cover: hedonic/utilitarian). (**A**) For purchasing decisions, participants indicated their willingness to pay a specific price for that book. (**B**) For perceptual choices, participants indicated the total number of people and lines on the book cover.

During the event-related fMRI sessions, participants performed either purchasing or perceptual decisions in alternating blocks (Fig. 1). Each good was presented twice: once during the purchasing decision and once during the perceptual decision. Before each block started, both blocks were cued visually so participants knew in which block they were performing. Hedonic and utilitarian goods were presented in a random order. There were four blocks, and each block had 76 stimuli.

To capture explicit hedonic/utilitarian values, we asked participants how much they would be willing to pay for the goods during purchasing decisions (willingness-to-pay: WTP), as previously described^11^. We considered the WTP scores as the economic value of goods, and the WTP scores for hedonic/utilitarian goods as hedonic/utilitarian values, respectively. We also assumed that participants automatically calculate WTP without the need for purchasing behavior during exposure to the goods and, therefore, WTP reflects the explicit and task-irrelevant economic values of the goods during purchasing decisions^25^.

We followed the Becker–Degroot–Marshak (BDM) auction method, which is widely used in economics to incentivize participants to reveal their true values for goods without actually providing all the selected goods^26^. In brief, participants were given money (450 yen in our case) for purchasing decisions and state their WTP for each item. They placed bids on the goods from a range of prices (0 yen, 150 yen, 300 yen or 450 yen in our case) as the stated WTP. At the end of the experiment, one trial was randomly selected, and then the experimenter generated a random selling price (from a known distribution of 0 yen, 150 yen, 300 yen, and 450 yen with equal probability in our case). If the random selling price was less than or equal to their stated WTP, the participant could purchase the goods at the random selling price and kept the remaining money. If the selling price was greater than their WTP, the participant could not purchase the item and keeps the entire 450 yen. Therefore, participants’ optimal strategy was to state their true value for goods on every trial.

Participants also performed perceptual decisions for a perceptual task. They viewed the cover of the book, and answered questions relating to perceptual levels: the total number of people and lines of the title in the book (by 2, 3, 4, or 5). Each book included at least one person and one line of title. They saw the same books during both decisions, but the order was pseudo-randomized to eliminate the order effects. Therefore, this procedure allowed motor responses and sensory input to be matched in both purchasing and perceptual tasks.

After MRI, participants viewed the same books and choose the reasoning for the purchase of each book from among four categories (hedonic: emotional wants, utilitarian: functional needs, both hedonic and utilitarian, and neither hedonic nor utilitarian). Participants also indicated their hedonic and utilitarian scores, their knowledge of content presented, and the brightness of the cover on a 7-point Likert scale.

The stimulus presentation and response recording during the experiments were controlled using Psychopy^27^.

### Measurements of reward sensitivity

We used the Japanese version of the BIS/BAS scales to assess reward sensitivity^28^. The Japanese version of the BIS/BAS scales consists of 20 items on a four-point Likert scale. BAS-Reward Responsiveness contains five items and both BAS-Drive and BAS-Fun Seeking consist of four items. The mean and SD were 16.33 ± 2.148 for BAS- Reward Responsiveness, 11.407 ± 2.227 for BAS- Fun Seeking, and 12.000 ± 1.881 for BAS-Drive. We did not observe any outliers (more than 3 SD) among these three BAS subscales.

### Behavioral analyses

To investigate the effects of hedonic and utilitarian attitudes on the value of goods, we estimated a linear mixed regression model. The dependent variable was hedonic/utilitarian value (the amount of money paid for each good) in a given trial. The independent variables were hedonic and utilitarian attitude scores indicated by the same participant for the goods after MRI, which were entered as fixed effects, whereas participants were modeled as random effects. We converted the range of prices (0 yen, 150 yen, 300 yen or 450 yen) to a range from 1 to 4 for the analyses.

To test for the differences in other factors between hedonic/utilitarian goods, the individual mean economic values, perceptual levels, and reaction times (RTs) were also compared using a paired *t*-test. We converted the range of perceptual levels (2, 3, 4, or 5) to a range from 1 to 4 for the analyses. We also checked the differences of confounding factors influencing values in hedonic and utilitarian goods in the post-MRI questions (the knowledge content, and brightness of the cover) using a paired *t*-test.

### Image acquisition

For all sessions, 41 transaxial gradient-echo images (echo time = 30 ms, flip angle = 90°, slice thickness = 3 mm, slice gap = 0.5 mm, field of view [FOV] = 192 mm, matrix = 64 × 64, voxel size = 3 × 3 × 3 mm) covering the entire cerebrum were acquired at a repetition time of 2.5 s using an echo planar sequence and an Achieva (3 T) MR scanner (Philips, Amsterdam, Netherlands). Slice orientation was acquired with a 30° tilt from the anterior commissure–posterior commissure to minimize signal loss in the orbitofrontal cortex^29^. To improve registrations, high-resolution T1-weighted structural images were also acquired (matrix = 240 × 240; repetition time = 6.5 ms; echo time = 3 ms; slice thickness = 1.0 mm; FOV = 24 cm; slices = 162).

### Image processing

The following preprocessing procedures and subsequent statistical analyses were performed using Statistical Parametric Mapping (SPM8) software (Wellcome Department of Imaging Neuroscience, London, UK) and MATLAB® (Mathworks Inc., Natick, MA, USA). These procedures included adjustment of acquisition timing across slices, correction for head motion, spatial normalization using the high-resolution T1-weighted structural imaging, and smoothing using a Gaussian kernel with a full width at half-maximum of 8 mm. The normalization step yielded BOLD images at 3 × 3 × 3 mm voxels.

### fMRI statistical analysis

To estimate BOLD signals with explicit and task-irrelevant WTP for hedonic and utilitarian goods, we ran general linear models (GLMs) at the first (individual) level. We ran separate GLMs for purchasing and perceptual decisions. For purchasing choices, we included two regressors (1, WTP; 2, perceptual levels) in the GLM. For perceptual choices, we include two regressors (1, WTP; 2, perceptual levels) in the other GLM. WTP was obtained during purchasing choices and, therefore, it was explicit during purchasing choices and task-irrelevant during perceptual choices. Perceptual levels were obtained during perceptual choices and, therefore, were task-irrelevant during purchasing choices and explicit during perceptual choices. The onset of the BOLD signal for the analysis occurred at the time of presentation of the book cover. Trial duration in the GLMs was set to the RTs on that trial. Each of the regressors was convolved with a canonical hemodynamic response function. The six rigid-body translation and rotation parameters derived from spatial realignment were also included as no-interests of the regressors. Data are high-pass filtered with a cutoff of 128 s.

For the second-level analysis, we constructed a 2 × 2 full-factorial design with four different conditions: (1) explicit hedonic values (EHV), (2) explicit utilitarian values (EUV), (3) task-irrelevant hedonic values (IHV) and (4) task-irrelevant utilitarian values (IUV). The factorial design included the images of the parameter estimates from the WTP parametric modulations for each condition. This model allowed us to test brain areas correlated with (1) EHV, (2) EUV, (3) IHV, and (4) IUV.

Second-level random-effects group contrast maps were investigated on these single-subject contrasts. The main effect of neural value representations and the correlation with WTP in each condition (EHV, EUV, IHV and IUV) were assessed.

We investigated the regions with common value representations for hedonic and utilitarian goods at explicit (EHV ∩ EUV) (H1). We performed a conjunction analysis^30^ based on a conjunction null hypothesis^30^. We performed the conjunction analysis assuming common value representations for hedonic and utilitarian goods for the explicit value judgments. Neural value-type specific representations were also assessed with explicit value representations, which were dependent on hedonic (EHV-EUV) and utilitarian values (EUV-EHV).

To test for regions showing common value representations for hedonic and utilitarian goods for task-irrelevant (IHV ∩ IUV) value judgments (H2), we performed the same analysis. We performed the same conjunction analysis that we did for common value representations for explicit hedonic and utilitarian goods. Neural value-type specific representations were assessed with task-irrelevant value representations dependent on hedonic values (IHV-IUV) and utilitarian values (IUV-IHV) (H3).

To test for modulation of value representations by BAS responsiveness, we created an interaction term for the unique effects of hedonic goods (EHV-UHV)-(EUV-IUV) and for the unique effects of task-irrelevant hedonic value (IHV-IUV)-(EHV-EUV) from the factorial design. Then, we investigated how individual differences in BAS modulate value representations of the interactions for the unique effects of hedonic value (H4), and the unique effects of task-irrelevant hedonic value, using a voxel-wise regression analysis. In the regression analysis, BAS values were entered as explanatory variables, contrasting the unique effects of the task-irrelevant hedonic value (EHV-UHV)-(EUV-IUV) and the hedonic value (IHV-IUV)-(EHV-EUV).

Activations were considered significant at *p* < 0.001 for the clusters that survived whole-brain or small volume correction (both FWE-corrected, *p* < 0.05^30–33^). For small volume correction, *a priori* areas were defined by 8 mm radius spheres around the activation peaks in the meta-analysis of valuation at the decision stage^13^: vmPFC (x = −2, y = 40, z = −8), left VS (x = −6, y = 8, z = −4), right VS (x = 6, y = 10, z = −8). The regions were constructed using the PickAtlas toolbox^34^.

## Results

### Behavioral data

#### Hedonic/utilitarian attitude scores for hedonic and utilitarian goods

To check the validity of hedonic and utilitarian goods for the neuroimaging samples, we tested for differences in hedonic/utilitarian attitude scores for hedonic and utilitarian goods in the post-MRI questions. Hedonic attitude scores are higher for hedonic goods than utilitarian goods (mean: 4.526 ± 0.653 for hedonic goods, mean: 2.392 ± 0.727 for utilitarian goods, t(26) = 12.387, p < 0.001), and utilitarian attitude scores are higher for utilitarian goods than hedonic goods (mean: 4.588 ± 0.765 for utilitarian, mean: 3.268 ± 0.743 for hedonic, t(26) = 11.596, p < 0.001). The results show that participants regard hedonic and utilitarian goods with a predominantly hedonic or utilitarian attitude, respectively.

#### Buying reasons for hedonic and utilitarian goods

Next, to confirm the different buying reasons for hedonic and utilitarian goods, we tested for differences in buying reasons using the post-MRI questions, following previous consumer literature^8^. For these analyses, we compared the differences in hedonic and utilitarian goods across participants using the paired *t*-test. Participants purchased hedonic goods based on hedonic reasons (emotional wants) for almost all trials (mean: 94.5 ± 5.19%.), and the proportion was higher for hedonic goods than for utilitarian goods (mean: 94.5 ± 5.19% for hedonic goods, mean: 1.40 ± 2.28% for utilitarian goods, t(26) = 87.200, p < 0.001). Participants purchased utilitarian goods based on utilitarian reasons (functional needs) for almost all trials (mean: 90.9 ± 8.73%), and the proportion was higher for utilitarian goods than for hedonic goods (mean: 90.9 ± 8.73% for utilitarian goods, mean: 2.25 ± 2.33% for hedonic goods, t(26) = 53.600, p < 0.001). The results indicate that hedonic and utilitarian goods are bought for distinct buying reasons based on either hedonic or utilitarian reasons, respectively, and that two value constructs exist.

#### Influences of hedonic and utilitarian attitudes on the value of goods

For a complementary approach, we estimated a linear mixed regression model to investigate the relationship between hedonic and utilitarian values (the amount of money paid for hedonic/utilitarian goods) and that between hedonic and utilitarian attitude scores. Linear mixed regression models show that hedonic attitude scores (β = 0.351, p = 0.015), but not utilitarian attitude scores (β = 0.031, p = 0.105), are significant predictors of hedonic values. In contrast to our expectations, hedonic (β = 0.217, p < 0.001) and utilitarian attitude scores (β = 0.522, p < 0.001) are found to be significant predictors of utilitarian values. The results suggest that hedonic attitudes influence both value types.

#### Mean economic values, perceptual levels and reaction times

We tested for differences in processing demands between both types of goods. Perceptual levels of hedonic and utilitarian goods did not significantly differ (t(26) = −0.065, p = 0.949, paired t test). The individual mean SV (subjective value) and PV (perceptual value) of hedonic and utilitarian goods were not significantly correlated, indicating that perceptual demands are independent of WTP for both goods (r = 0.060, p = 0.762, for hedonic goods; r = 0.055, p = 0.761 for utilitarian goods). RTs for hedonic and utilitarian goods purchasing choices did not significantly differ (t(26) = −0.442, p = 0.662, paired *t*-test). Perceptual choice RTs for hedonic and utilitarian goods were also not significantly different (t(26) = −1.779, p = 0.087, paired *t*-test). However, significant differences were observed for RTs for purchasing and perceptual hedonic goods (*t*(26) = −3.594, *p* = 0.001, paired *t*-test) as well as RTs for purchasing and perceptual utilitarian goods] (*t*(26) = −3.240, *p* = 0.003, paired *t*-test). Several characteristics of hedonic and utilitarian goods did not differ, including knowledge content scores (*t*(26) = 1.775, *p* = 0.078, paired *t*-test) and brightness score for the book cover scores (*t*(26) = 1.377, *p* = 0.171, paired *t*-test). These results indicate that the goods (hedonic vs. utilitarian) did not have different processing demands, including PV or RTs, for purchasing and perceptual choices. In contrast, task types (purchasing vs. perceptual) had different processing demands.

These consumer attitude results suggest that participants purchased hedonic and utilitarian goods based on different reasons, and that we can regard each value (the amount of money paid for each good) as hedonic and utilitarian, respectively. The other behavioral results also indicate that both types of goods are based on identical motor responses and perceptual levels and, therefore, allow for an unbiased comparative analysis of how hedonic and utilitarian values are represented in the brain in both explicit and task-irrelevant manners.

#### Neural value representations of hedonic and utilitarian goods for explicit judgment

**Figure 2:**
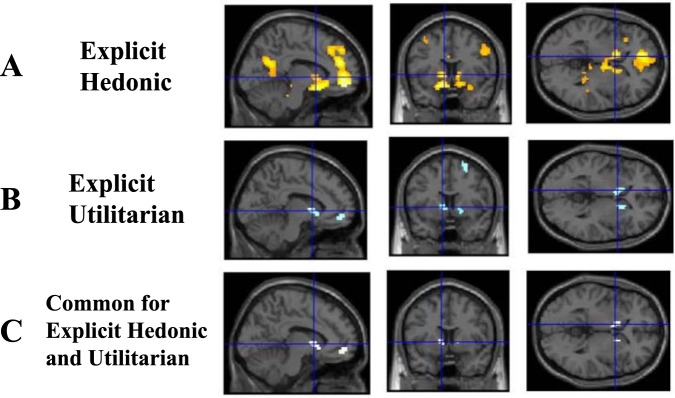
Brain regions correlated with hedonic and utilitarian values during purchasing decisions. (**A**) Voxels in pale pink correlated with hedonic values, (**B**) voxels in blue correlated with utilitarian values, and (**C**) the conjunction of hedonic and utilitarian values shown in yellow. Sagittal, coronal, and axial sections showing the peak activation of vmPFC (x = −6, y = 47, z = −8), right VS (x = 9, y = 14, z = −5), and left VS (x = −9, y = 8, z = 1), respectively. The peak activations derived from the conjunction analysis of hedonic and utilitarian values during purchasing decisions. The data are presented in figures at a threshold of p < 0.001, uncorrected for illustrative purposes only. Coordinates are reported in the stereotaxic space of the MNI. Abbreviations: vmPFC (ventromedial prefrontal cortex); VS (ventral striatum); MNI (Montreal Neurological Institute).

To test for regions showing common responses to explicit values for both hedonic and utilitarian goods (H1), we performed a conjunction analysis. The small-volume-based analysis revealed significant common neural activation in the bilateral VS regions (Fig. 2). These results were consistent with the neural common currency hypothesis. The results are shown in Table 1.

**Table 1:** Neural value-type common representations.

Regions	Local max				*K*	*P*
	*x*	*y*	*z*	*Z*		
**Explicit economic values independent of hedonic and utilitarian values (EHV ∩ EUV)**						
vmPFC (SVC)	n.s.					
Left VS (SVC)	−9	8	1	3.76	17	0.0002
**Task-irrelevant economic values independent of hedonic and utilitarian values (IHV ∩ IUV)**						
	n.s.					

Brain regions showing common value representations for hedonic and utilitarian goods at explicit (EHV ∩ EUV) and task-irrelevant (IHV ∩ IUV) value judgments.A conjunction analysis based on a conjunction null hypothesis. The uncorrected statistical threshold was p < 0.001, which was corrected to p < 0.05 using cluster size. Coordinates are reported in the stereotaxic space of the MNI.

Abbreviations: vmPFC (ventromedial prefrontal cortex); VS (ventral striatum); SVC (small volume correction); EUV (explicit utilitarian values); IHV (task-irrelevant hedonic values); EHV (explicit hedonic values); IUV (task-irrelevant utilitarian values); n.s. (non-significant); MNI (Montreal Neurological Institute).

We then investigated the neural value representation for both explicit and hedonic values. The whole brain analysis revealed that EHV was positively correlated with the activation of broad vmPFC and VS regions as well as the posterior cingulate cortex (PCC). The small-volume-based analysis also showed significant neural activation of EHV and EUV in the vmPFC and VS regions. However, we did not find any significant differences in explicit values between hedonic and utilitarian goods. The results are shown in Table 2.

**Table 2:** Neural value representations.

Regions	Local max				*K*	*P*
	*x*	*y*	*z*	*Z*		
**EHV**						
Orbitofrontal cortex	−3	56	−8	5.84	1122	<0.001
Medial prefrontal cortex	−9	44	22	5.16	—	—
Left caudate	−12	11	−14	5.31	1252	<0.001
Right precuneus	−3	−64	19	4.36	207	0.0005
vmPFC (SVC)	−6	47	−5	4.64	37	<0.001
Left str (SVC)	−12	8	1	4.74	35	<0.001
Right str (SVC)	9	14	−5	3.91	32	0.0001
vmPFC (SVC)	−6	47	−8	3.14	1	0.0016
Left VS (SVC)	−6	5	1	3.76	22	0.002
Right VS (SVC)	12	14	−8	3.81	20	0.0002
**IHV**						
	n.s.					

Brain areas showing positive correlation with the amount of money subjects were willing to pay during the purchasing task (explicit values) and perceptual tasks (task-irrelevant values). The uncorrected statistical threshold was p < 0.001, which was corrected to p < 0.05 using cluster size. Coordinates are reported in the stereotaxic space of the MNI.

Abbreviations: vmPFC (ventromedial prefrontal cortex); VS (ventral striatum); SVC (small volume correction); EUV (explicit utilitarian values); IHV (task-irrelevant hedonic values); EHV (explicit hedonic values); IUV (task-irrelevant utilitarian values); MNI (Montreal Neurological Institute).

#### Neural value representations of hedonic and utilitarian goods with task-irrelevant judgments

To test for regions showing common responses to task-irrelevant values for both hedonic and utilitarian goods (H2), we performed a conjunction analysis. However, we did not find any task-irrelevant economic values in common between hedonic and utilitarian values. Therefore, the results do not support H2. When testing for regions dependent on task-irrelevant hedonic (vs. utilitarian) values, the whole brain analysis found that the task-irrelevant hedonic (vs. utilitarian) values show more activity in the occipito-temporal regions including the lingual gyrus, fusiform gyrus, and parahippocampal gyrus. Small-volume-based analysis revealed that task-irrelevant hedonic (vs. utilitarian) values show more activity in the vmPFC. However, the value type (hedonic/utilitarian) by judgment mode (explicit/task-irrelevant) interaction was not significant, indicating that the significance of the mere contrast between task-irrelevant hedonic and utilitarian values may not be caused by the interactions. The results do not support H3. We did not find any significant differences for task-irrelevant economic values that are dependent on utilitarian values. The results are shown in Table 3.

**Table 3:** Neural value-type specific representation.

Regions	Local max				*K*	*P*
	*x*	*y*	*z*	*Z*		
**Explicit economic values dependent on hedonic values (EHV - EUV)**						
	n.s.					
**Explicit economic values dependent on utilitarian values (EUV - EHV)**						
**Task-irrelevant economic values dependent on hedonic values (IHV - IUV)**						
vmPFC (SVC)	3	44	−5	3.28	5	0.0010
**Task-irrelevant economic values dependent on utilitarian values (IUV - IHV)**						
	n.s.					

Brain areas showing explicit value representations dependent on hedonic values (EHV-EUV) and utilitarian values (EUV-EHV), and task-irrelevant value representations dependent on hedonic values (IHV-IUV) and utilitarian values (IUV-IHV). The uncorrected threshold was uncorrected p < 0.0001, and was corrected to p < 0.05 using cluster size. Coordinates are reported in the stereotaxic space of the MNI.

Abbreviations: vmPFC (ventromedial prefrontal cortex); VS (ventral striatum); SVC (small volume correction); EUV (explicit utilitarian values); IHV (task-irrelevant hedonic values); EHV (explicit hedonic values); IUV (task-irrelevant utilitarian values); n.s. (non-significant); MNI (Montreal Neurological Institute).

Then, we investigating neural value representation for each task-irrelevant hedonic and utilitarian value. The whole brain analysis found that IHV was associated with greater activity in the orbitfrontal cortex, and small-volume-based analysis revealed that IHV was associated with greater activities in vmPFC. However, the results for IUV (see Table 2) were not significant.

#### BAS modulating neural value representations of hedonic and utilitarian goods

We did not find any relationships between BAS (total scores and subscales) and value representation dependent on task-irrelevant hedonic value (IHV-IUV)-(EHV-EUV) in the whole brain or in the SVC analyses. We also did not find any relationships between BAS (total scores and subscales) and value representation dependent on explicit hedonic value (EHV + IHV) − (EUV + IUV) in the whole brain or in the ROI analyses.

For an exploratory analysis, we investigated whether the vmPFC activity of IHV modulated BAS. We defined the ROI within the vmPFC that encompassed voxels above our significance threshold during the task-irrelevant value judgment of hedonic value. MarsBaR was used to extract the average parameter estimates within the ROI. However, the vmPFC activity was not related to BAS (r = −0.035, p = 0.862). Therefore, the results did not support H4.

## General Discussion

The aim of this study was to understand the relationship between affect and task-irrelevant value judgments in the brain. Although hedonic and utilitarian goods are purchased based on different buying considerations, there was no direct evidence regarding whether (1) the value of hedonic goods was commonly processed in the value-related regions during purchasing decisions (H1) and/or during value-irrelevant judgments (H2). Additionally, we did not find support for the hypothesis that the task-irrelevant value of hedonic (vs. utilitarian) goods was amplified (H3), or for the hypothesis that the task-irrelevant value of hedonic (vs. utilitarian) goods was amplified specifically in reward-sensitive consumers (H4). Neural data can help address these questions and refine our understanding of how consumers represent the value of consumer goods. Although the results did not support H2-4, the findings support H1. Value of hedonic and utilitarian goods were represented in the ventral striatum during purchasing. Together, the findings provide theoretical insights into how consumers represent the value of goods bought by different reasons.

Values of hedonic and utilitarian goods are similarly processed in the ventral striatum during purchasing. Although neuroscience research has shown that common brain regions are involved in a variety of types of valuation^1,13^, it remains unclear from consumer research^35^ whether hedonic and utilitarian goods are evaluated on a single dimension or multiple dimensions. The current findings support a unidimensional value model, and refine the consumer value model.

The mechanism underlying the unidimensionality of valuations of hedonic and utilitarian goods during purchasing may be positive affect; that is, the value of both types of goods may be represented as positive affect (VS). Behavioral data also support the idea that the values of both hedonic and utilitarian goods are derived from positive affect. Only hedonic attitudes (affective states) influence values for both hedonic and utilitarian goods. Although people explained that they bought utilitarian goods for utilitarian reasons, evaluations of utility may automatically result in various affective states in the brain, and subjective reports may not capture this transformation. Although our result accords with a uni-dimensional model, the underlying mechanisms differ from the conventional model. Traditionally, the uni-dimensional model has considered the underlying mechanisms of valuation cognitive rather than affective^35^. Our findings underscore the relevance of positive affect to value-based consumer decision making across different types of products.

We did not find evidence of task-irrelevant hedonic (vs. utilitarian) values or of modulation of evaluations by individual differences in reward sensitivity. Although it seems possible that the perceptual task in this study may not be appropriate for capturing task-irrelevant value, we observed task-irrelevant hedonic value in the vmPFC. Moreover, the task demandingness (measured by RTs) of judgments of hedonic and utilitarian goods did not differ for either explicit or perceptual judgments. Thus, task appropriateness and task demandingness might not have contributed to the results. However, behavioral evidence showed that value of utilitarian products was derived from positive affect, which may prevent us from detecting task-irrelevant hedonic (vs. utilitarian) values in the brain. Further study should use a more sophisticated experimental design to separate the effects of the hedonic and utilitarian dimensions on value.

The study has limitations. First, all participants were university students, and the gender ratio was unbalanced (6 females out of 27 total participants). Further studies should test whether these results are consistent in a variety of populations with balanced gender ratios. Second, task types (purchasing vs. perceptual) had different processing demands, or RTs. This might explain why we did not find any relationships between (total or subscale) BAS scores and value representation as a function of task-irrelevant hedonic value (IHV-IUV)-(EHV-EUV).

In conclusion, this study revealed, using fMRI, how consumers value different types of products during explicit and task-irrelevant judgment. The results showed that the values of hedonic and utilitarian goods were both processed in the same brain regions (ventral striatum and ventromedial PFC) during purchasing. This finding supports and reinforces the neural common currency hypothesis, and provides theoretical insight into constructs of consumer value representation.

## Data Availability

The MRI datasets generated and/or analyzed in the present study are not publicly available because the informed consent process does not allow for it. However, the data are available from the corresponding author for reasonable requests.
