# Author Correction: Common neural value representations of hedonic and utilitarian products in the ventral striatum: An fMRI study

**DOI:** 10.1038/s41598-020-58293-z

**Published:** 2020-01-24

**Authors:** Kosuke Motoki, Motoaki Sugiura, Ryuta Kawashima

**Affiliations:** 1 Department of Food Management, Miyagi University, Sendai, Japan; 2 Institute of Development, Aging and Cancer, Tohoku University, Sendai, Japan

Correction to: *Scientific Reports* 10.1038/s41598-019-52159-9, published online 30 October 2019

The original version of this Article contained an error in the title of the paper, where the word “Striatum” was incorrectly given as “Stratum”. This has now been corrected in the PDF and HTML versions of the Article.

